# The Immunomodulatory Activity of Meningococcal Lipoprotein Ag473 Depends on the Conformation Made up of the Lipid and Protein Moieties

**DOI:** 10.1371/journal.pone.0040873

**Published:** 2012-07-23

**Authors:** Ching-Liang Chu, Yen-Ling Yu, Yueh-Chen Kung, Pei-Yu Liao, Ko-Jiunn Liu, Yen-Tzu Tseng, Yuan-Chuen Lin, Steve Shih-Yang Hsieh, Pele Choi-Sing Chong, Chiou-Ying Yang

**Affiliations:** 1 Graduate Institute of Immunology, College of Medicine, National Taiwan University, Taipei, Taiwan; 2 Vaccine Research and Development Center, National Health Research Institutes, Miaoli, Taiwan; 3 Institute of Molecular Biology, National Chung Hsing University, Taichung, Taiwan; 4 National Institute of Cancer Research, National Health Research Institutes, Tainan, Taiwan; 5 School of Medical Laboratory Science and Biotechnology, Taipei Medical University, Taipei, Taiwan; Universite de la Mediterranee, France

## Abstract

We have previously demonstrated that the meningococcal antigen Ag473 in the presence of Freund’s adjuvant can elicit protective immune responses in mouse challenge model. In this study, we evaluated the structural requirement for the immunological activity and the possible signaling pathway of recombinant Ag473 antigen produced in *E. coli*. We found that lipidated Ag473 (L-Ag473) possesses an intrinsic adjuvant activity that could be attributed to its ability to activate dendritic cells and promote their maturation. In addition, we found that L-Ag473 can activate human monocytes and promote maturation of human monocyte-derived dendritic cells. These results provide an indirect support that L-Ag473 may also be immunogenic in human. Interestingly, the observed activity is dependent on the overall conformation of L-Ag473 because heating and proteinase K treatment can diminish and abolish the activity. Furthermore, our data suggest a species-differential TLR recognition of L-Ag473. Overall, these data suggest a new paradigm for the ligand-TLR interaction in addition to demonstrating the self-adjuvanting activity of the vaccine candidate L-Ag473.

## Introduction

*Neisseria meningitidis* (NM) is a capsulated Gram-negative bacterium causing meningitis and septicemia, life-threatening invasive infections especially in children and young adults. Currently, capsular polysaccharide (CPS)-based vaccines for protection against serogroups A, C, Y, and W135 are commercially available. Because of molecular structure identical to the serogroup B CPS is present in human fetal neural tissue; it is poorly immunogenic in humans [1]. The development of serogroup B meningococcal vaccines is thus focused primarily on the outer-membrane components. This field has been progressing rapidly after completion of the MC58 genome project [2]. Among the identified vaccine candidates, many of them are lipoproteins [3].

According to the principle of vaccine, activation of antigen-presenting cells (APCs) is required for inducing the protective immune responses. Dendritic cells (DCs) are professional APCs which are critical in the initiation of immune responses during infection or vaccination [4]. They capture antigens and then migrate to secondary lymphoid organs where they present processed antigens to stimulate antigen-specific T-cells [5], [6], [7]. DCs become mature after stimulating by typical microbial molecules or pathogen-associated molecular patterns (PAMPs) and enhance the ability of activating T cells [8], [9], [10]. Toll-like receptors (TLRs) are the most important pattern recognition receptors (PRRs) to detect PAMPs in DCs [11]. TLR ligand induces the release of both cytokines and chemokines and the up-regulation of MHC class II and costimulatory molecule expression [12], [13].

Bacterial lipoproteins are a group of proteins that are functionally diverse and can initiate many effects on immune cells such as activation of macrophages and DCs resulting in the release of cytokines and DC maturation [14], [15]. These immunomodulating activities are mediated primarily by TLR2 in cooperating with TLR1 or TLR6 depending on whether the N-terminal cysteine is triacylated or diacylated [16], [17]. However, lipoproteins act via both TLR2/TLR4 and TLR4 alone have been reported [18], [19], [20].

Although several meningococcal lipoproteins have been identified, very few have been characterized further. Ag473 recently identified in our laboratory is a surface lipoprotein (∼10 kDa) containing 7-amino acid tandem repeats [21]. Both diacylated and triacylated isoforms of recombinant Ag473 were expressed in the C43(DE3) *E. coli* strain and preliminary immunological study indicated that both isoforms are capable of upregulating cytokines expression in THP-1 cells [22]. The potential to use Ag473 as a vaccine against serogroup B meningococci has been demonstrated by the observation that active immunization with one variant in the presence of adjuvant conferred protection against a heterologous meningococcal strain [21]. The adjuvant activity of the N-terminal lipopeptide of Ag473 has been evidenced by a lipidated viral protein encoded by the recombinant gene fused with the first 40 codons of the *ag473* gene [23]. Since the mature Ag473 consists of only 12 kinds of amino acids with no aromatic amino acids (accession: AAT67224-227) and rich in alanine that is of interest to know whether the protein conformation plays any roles in the adjuvant activity. In the present study, we evaluate the structural requirement for the immunopotentiating activity of recombinant Ag473 produced in *E. coli* and identify its possible signaling pathway in the immune responses.

**Figure 1:**
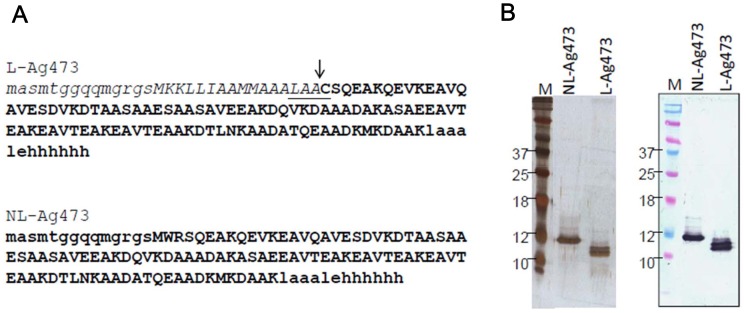
Sequences and purification of the recombinant proteins. (A) Amino acid sequences derived from the insert DNA are capitalized, lipobox is underlined, the cleavage site is indicated by an arrow, and amino acid sequences of mature proteins are bolded. (B) Purified recombinant lipidated (L-Ag473) and non-lipidated (NL-Ag473) Ag473 in PBS, were run on a 15% SDS-polyacrylamide gels and subjected to silver staining or Western blotting (right panel).

**Figure 2:**
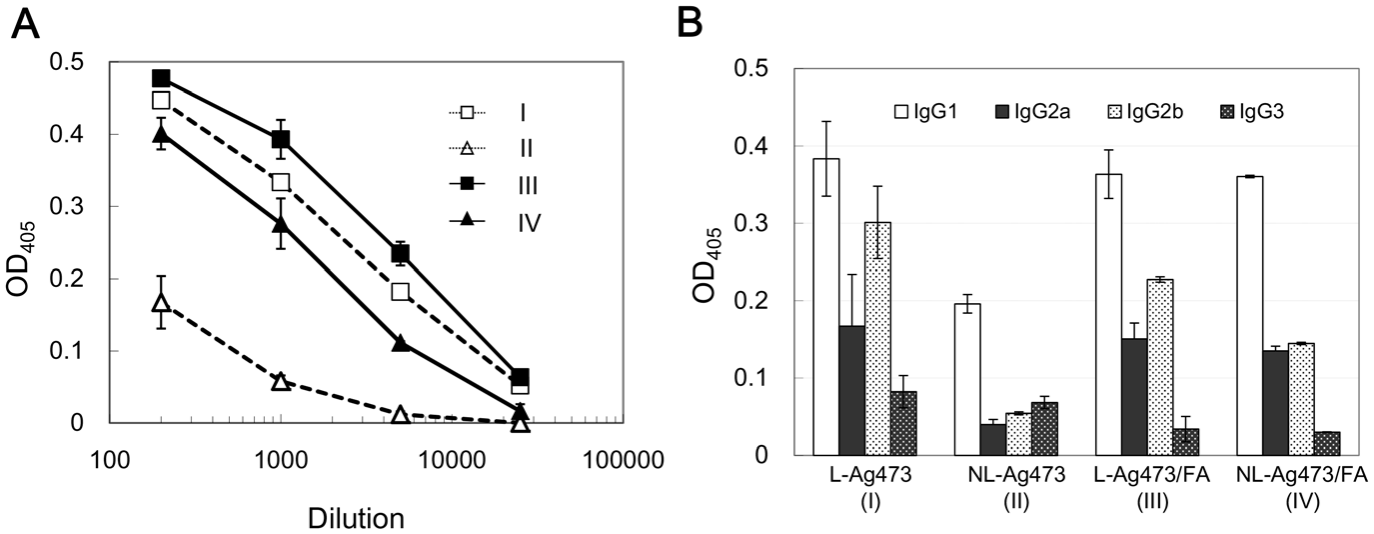
Promotion of specific antibodies by Ag473 immunization. Antibody titers (A) and the IgG isotype profiles (B) of the Ag473-immune sera determined by ELISA. (A) AP- anti-mouse Ig (μγα) was used as the secondary antibody; (B) anti-NL-Ag473 was 100X diluted while others were 1000X. The shown readings were obtained by subtracting the readings of preimmune sera from that of immune sera.

## Materials and Methods

### Expression and Purification of Recombinant Proteins

Recombinant Ag473 proteins were produced in E. coli using expression vector pET21. Plasmid pET21-Ag473/NMB1468 [21] was used to produce the lipidated Ag473 (L-Ag473). The gene coding for non-lipidated Ag473 with Trp-Arg dipeptide on the N-terminus (NL-Ag473) was PCR amplified from the expression plasmid pET21-Ag473/NMB1468 using primers GGATCCATGTGGCGCTCGCAAGAAGCCAAACAGGAG and AAGCTTGGCGGCATCTTTCATTTTGTCTGC. The resultant DNA fragment was inserted into the BamHI-Hind III sites of the pET21 to generate pET21-NL-Ag473 plasmid. Recombinant proteins were expressed as described previously [21] and purified by monoclonal antibody 4-7-3 affinity chromatography. Briefly, bacteria were cultured at 28°C for 18 hours in the presence of IPTG (1 mM) and harvested by centrifugation. The bacterial pellets were suspended in PBS containing 0.5% Triton X-100 and subjected to sonication. Unbroken cells and debris were removed by centrifugation (Sorvall RC5C SS-34 rotor, 12000 rpm) for 30 min at 4°C. The supernatants were passed through a 0.45 µM filter and loaded to the 4-7-3 affinity column which was prepared by coupling the Protein-A-Sepharose purified 4-7-3 to CNBr-activated Sepharose™ 4B beads according to the manufacturer’s instructions (71-7086-00 AF, GE Healthcare). The column was washed extensively with 50 mM Tris-HCl (pH 7) followed by 30% ethylene glycol/PBS, and then eluted with 0.5 M NaCl in acetate buffer (pH 4.0), 0.1 M NaCl/0.5% NP-40/PBS (low salt), and 1M NaCl/0.5% NP-40/PBS (high salt). The purity of recombinant proteins in each fraction was monitored by SDS-PAGE followed by silver staining and the antigenicity of the recombinant proteins was examined by Western blotting as previously described [21]. High salt eluate was dialyzed against PBS and the concentration of purified NL-Ag473 was determined by measuring the absorption at 280 nm (1 OD  = 2.4 mg/ml) and used as the standard to estimate the concentration of L-Ag473 by comparing the band intensity on a silver stained gel.

**Figure 3:**
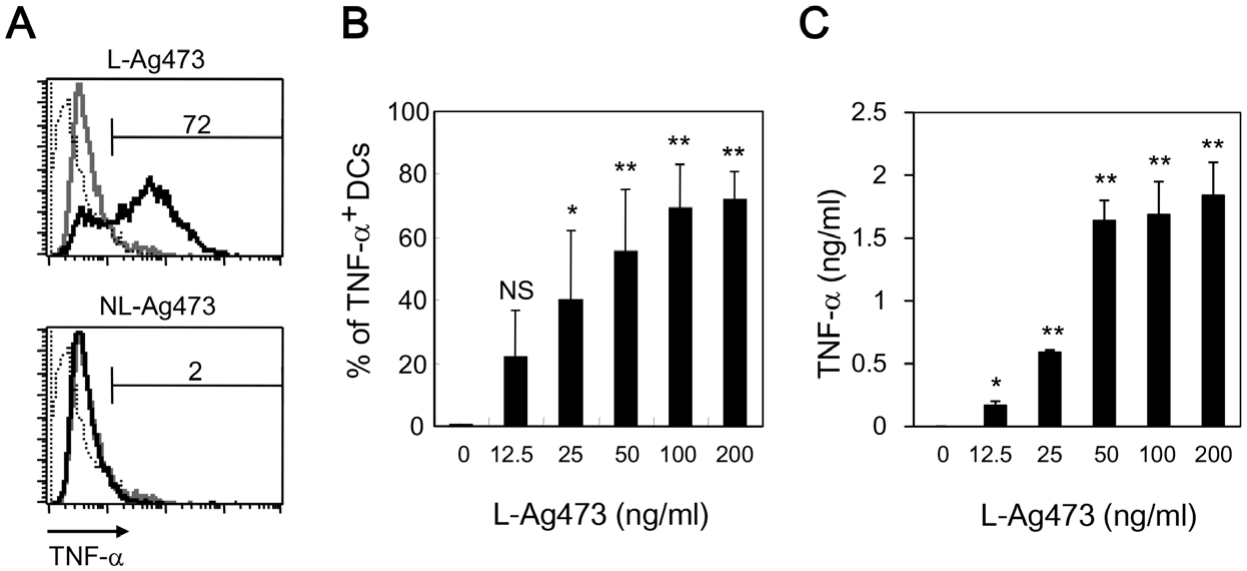
L-Ag473, but not NL-Ag473 activates BMDCs and macrophages. (A) BMDCs were treated with L-Ag473 or NL-Ag473 (100 ng/ml) (black line) for 6 hours and the intracellular TNF-α of CD11c^+^ cells were determined by flow cytometry. Dotted and grey lines represent the isotype-matched control Ab and untreated cell, respectively. (B, C) Dose-dependent response of BMDC (B) and macrophages (C). ^NS^ *p*>0.05; *p<0.05; **p<0.01 (Student’s *t*-test), comparing Ag473-treated to non-treated cells. All data are representative of two to four independent experiments.

### Immunization and Evaluation of Antibody Responses

All mice were housed in the barrier facility in National Chung Hsing University, Taichung, Taiwan, under an Institutional Animal Care and Use Committee-approved protocol. Eight week-old Balb/c mice were immunized intraperitoneally with L-Ag473 or NL-Ag473 four times at two-week intervals. Each dose consisted of 7.5 µg of purified protein without or with Freund’s adjuvant (FA; complete Freund’s adjuvant for the first injection and incomplete Freund’s adjuvant for boosting) in a volume of 200 µl. The antibody response was monitored by ELISA in 96-well plates coated with Ag473 (0.5 µg/well) essentially as previously described [21]. The relative amounts of specific IgG subclasses bound to the immobilized NL-Ag473 were determined using the isotyping kit from Sigma. The reading of specific antibodies was obtained by subtracted the reading of preimmune sera from the antisera.

**Figure 4:**
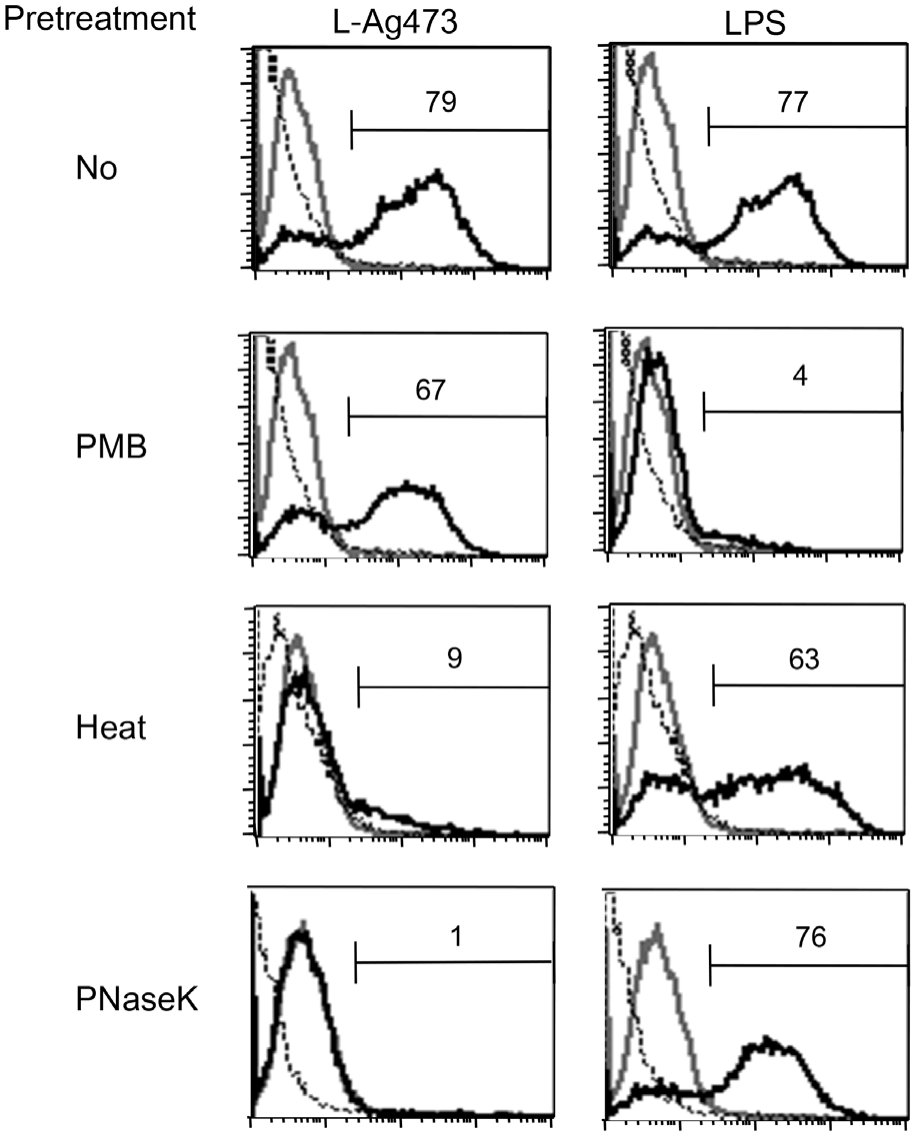
The biological activity of L-Ag473 is retained after pretreatment with PMB but abolished by heat or proteinase K treatment. BMDCs were treated with L-Ag473 (100 ng/ml) or LPS (20 ng/ml) for 6 hours and the intracellular TNF-α of CD11c^+^ cells were determined by flow cytometry. Dotted and grey lines represent the isotype-matched control Ab and untreated cell, respectively. PNaseK, proteinase K.

### ELISA

The microtiter plates were coated with 50 µl of Ag473 (10 µg/ml). After blocking with 2% nonfat milk in PBS for 2 hours and rinsed three times with PBS, the test antiserum (50 µl) was added to each well. For the antibody titer experiments, alkaline phosphatase conjugated anti-mouse Ig (μγα) was used as the secondary antibody. The Ag473 specific IgG subclasses were determined using 1000-fold diluted antisera with the exception of the anti-NL-Ag473 (100-fold dilution). The Ag473-bound IgG was detected by goat anti-mouse isotype specific antibody followed by alkaline phosphatase conjugated rabbit anti-goat IgG, and then developed by incubation with *p*-nitrophenyl phosphate substrate (Sigma) in diethanolamine buffer (pH 9.8) for 1 hour in the dark at room temperature. The absorbance at 405 nm was read in a Bio-Kinetic Reader EL 312e (Bio-Tek Instrument Inc., Winooske, VT).

**Figure 5:**
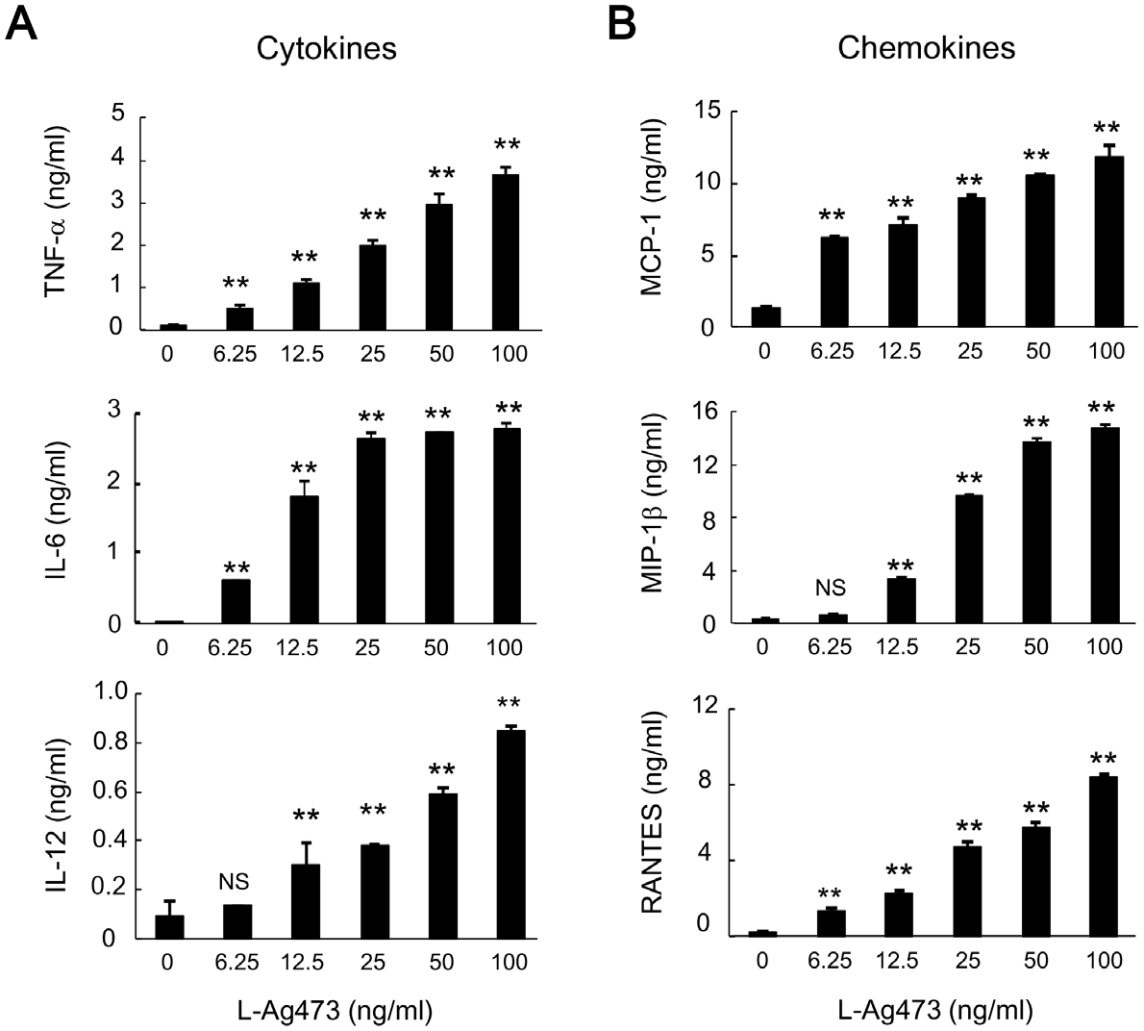
L-Ag473 induces cytokine and chemokine production by BMDCs. BMDCs were incubated with L-Ag473 for 24 hours (or 6 hours for TNF-α and RANTES). Supernatants were collected and (A) TNF-α, IL-6, and IL-12; (B) MCP-1, MIP-1, and RANTES were determined by ELISA. Data are shown as mean ± SD from triplicate DC cultures; ^NS^ *p*>0.05; *p<0.05; **p<0.01 (Student’s *t*-test), comparing Ag473-treated to non-treated cells. All data are representative of three independent experiments.

**Figure 6:**
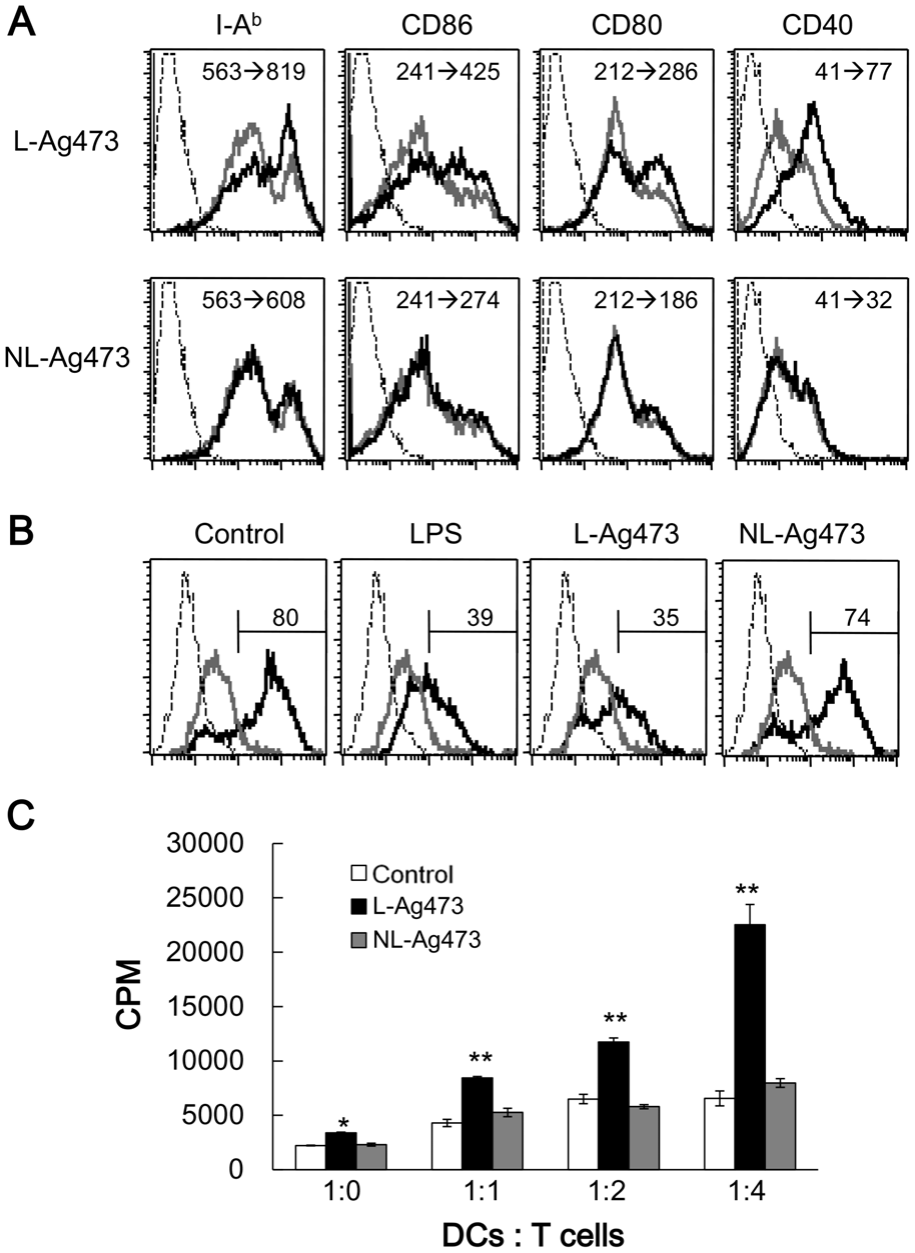
L-Ag473 promotes BMDC maturation and DC-induced T cell activation. (A) BMDCs were treated with L-Ag473, NL-Ag473 (100 ng/ml) (black line), or untreated (gray line) for 16 hours, and then stained with anti-I-A^b^, CD86, CD80, and CD40 mAbs. Dotted line represents staining with an isotype-matched control Ab. The change of mean fluorescence intensity (MFI) from no treatment to Ag473 treatment is shown on each picture. (B) BMDCs were treated with LPS (20 ng/ml), L-Ag473, NL-Ag473 (100 ng/ml), or untreated for 16 hours. The ability of endocytosis of DCs was determined by the uptake of dextran-FITC at 4°C (gray line) or 37°C (black line). Dotted line represents untreated DCs without dextran-FITC. The percentages of dextran-FITC^+^CD11c^+^ cells were shown above the regional markers. All data are representative of two to four independent experiments. (C) CD4^+^ OT-II T cells were isolated and co-cultured with L-Ag473 or NL-Ag473 (100 ng/ml)-activated DCs pulsed with OVA peptide (2 µg/ml) at indicated ratio of DC: T cell for 72 hours. T cell proliferation was determined by [^3^H]thymidine incorporation. Data are shown as mean + SD from triplicate DC cultures; *p<0.05; **p<0.01 (Student’s *t*-test), comparing L-Ag473-treated to non-treated cells.

### DC and Macrophage Preparation

Mice were housed in the barrier facility in National Health Research Institutes, Miaoli, Taiwan, under an Institutional Animal Care and Use Committee-approved protocol. Bone marrow-derived DCs (BMDC) or macrophages were generated from C57BL/6 mice (NLAC, Taipei, Taiwan) as previously described [24], [25]. OT-II T cell transgenic and MyD88^−/−^ mice were provided by Dr. Anthony DeFranco (UCSF, CA). TLR2^−/−^ mice were supported by Dr. John Kung (Academia Sinica, Taiwan). C3H/HeOuJ (WT) and C3H/HeJ (TLR4 mutant) mice were gifts from Dr. Zaodung Ling (NHRI, Taiwan). Human monocyte-derived DCs (MoDCs) were generated from peripheral blood as described previously [26]. Peripheral blood mononuclear cells (PBMCs) from healthy donors were obtained from the Taiwan Blood Services Foundation with the approval of Institutional Review Board.

**Figure 7:**
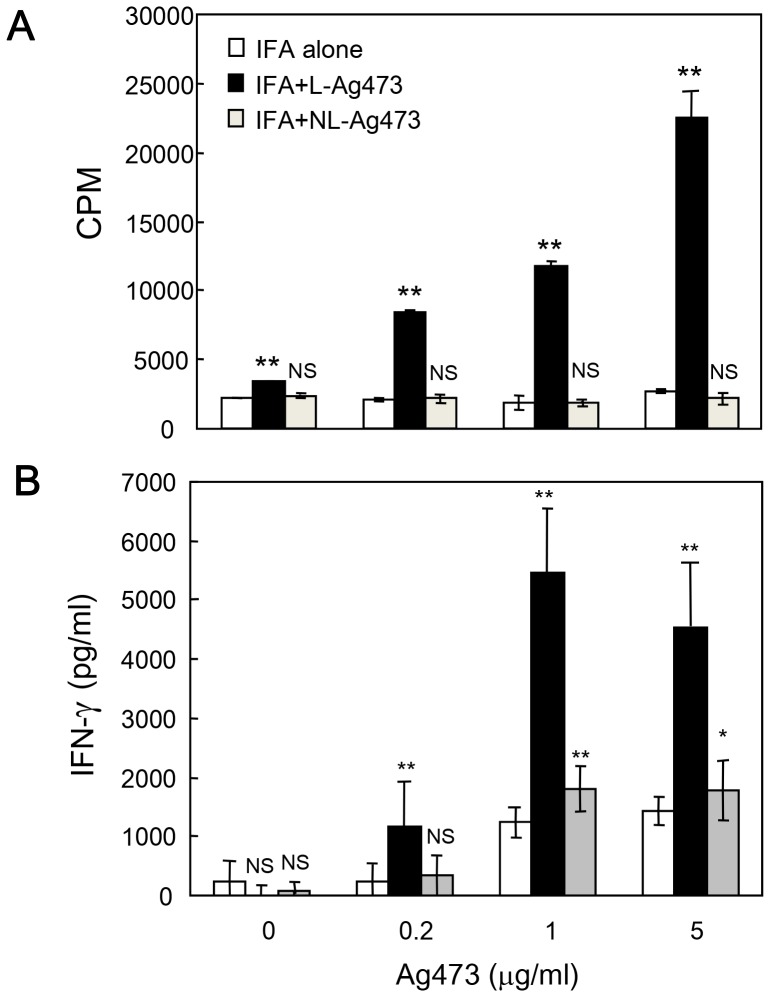
Induction of specific T cell activation by Ag473 immunization in recall assay. C57BL/6 mice were immunized with IFA alone, IFA + L-Ag473 or NL-Ag473 (10 µg) via footpad injection. Draining lymph node cells were collected after 10 days and cultured in 96-well plates with indicated concentration of Ag473 for 3 days. (A) T cell proliferation was determined by [^3^H]thymidine incorporation. (B) IFN-γ production was measured by ELISA. Data are shown as mean + SD from triplicate cultures; ^NS^ *p*>0.05; *p<0.05; **p<0.01 (Student’s *t*-test), comparing L-Ag473/NL-Ag473-immunized to control mice. All data are representative of two to three independent experiments.

### DC Maturation

As described previously [27], DCs cultured in 24-well plates at day 6 were treated with LPS (20 ng/ml, Sigma-Aldrich), Pam_2_CSK_4_ (50 ng/ml), CpG (500 µM, Invivogen), and various recombinant Ag473 proteins (100 ng/ml) for 16 hours. After blocked with anti-CD16/CD32 mAb 2.4G2 (BD Pharmingen), the cells were stained with mAbs against CD11c, CD40, CD80, CD86, and I-A^b^ (Biolegend), and then analyzed by flow cytometry (FACSCalibur and Cell Quest software). For endocytosis assay, the treated or untreated DCs were incubated with 200 µg/ml Dextran-FITC (MW ∼77 kDa, Sigma-Aldrich) for 1 hour at 4°C or 37°C. Cells were washed with cold PBS, stained with anti-CD11c Ab, and then analyzed by flow cytometry.

**Figure 8:**
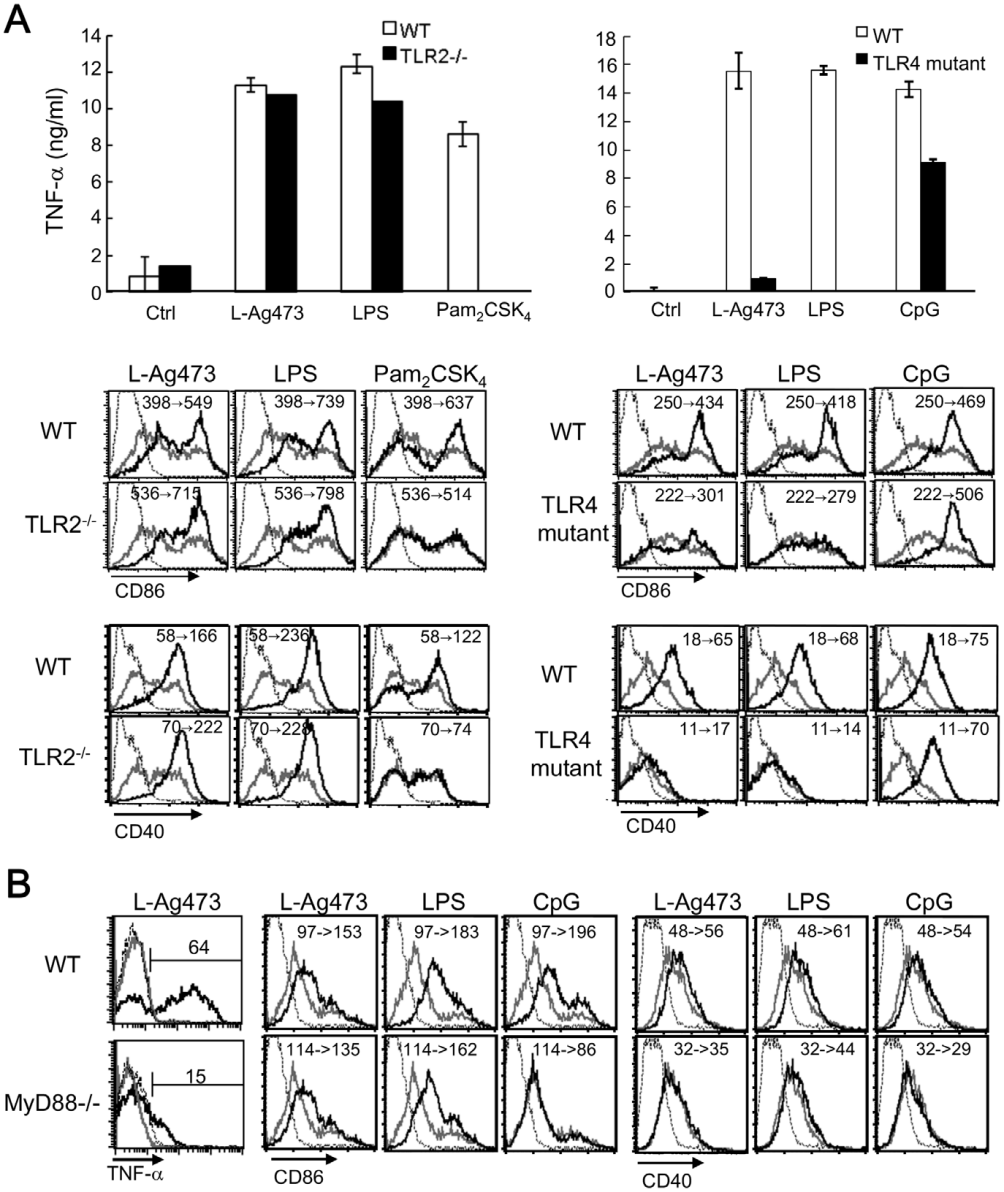
TLR4 was involved in the L-Ag473-induced BMDC activation. DCs derived from C57BL6 (B6 WT), TLR2^−/−^, C3H/HeOuJ (C3H WT), C3H/HeJ (TLR4 mutant) (A) and MyD88^−/−^ (B) mice were treated with L-Ag473 (100 ng/ml), LPS (20 ng/ml), Pam_2_CSK_4_ (50 ng/ml), or CpG DNA (100 nM). (A) TNF-α production was measured by ELISA. Data are shown as mean ± SD from triplicate DC cultures; ^NS^ *p*>0.05; *p<0.05; **p<0.01 (Student’s *t*-test), comparing TLR2^−/−^/TLR4 mutant to WT cells. (B) Maturation was determined by flow cytometry. Cells were treated with L-Ag473, LPS, Pam_2_CSK_4_, or CpG DNA (black line) or untreated (gray line) for 16 hours. Dotted line represents staining with an isotype-matched control Ab. The change of MFI from no treatment to stimuli treatment is shown on each picture. The expression of CD86 and CD40 was shown.

**Figure 9:**
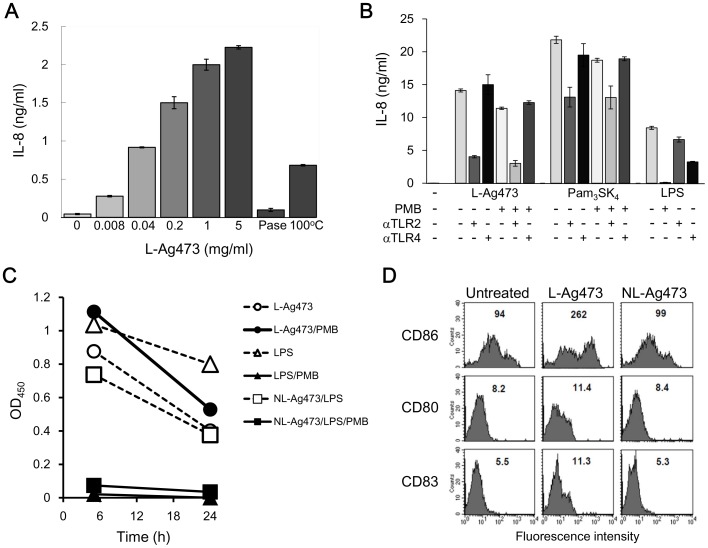
The effect of L-Ag473 on human cells. (A) THP-1 cells (2×10^5^/well) were incubated with the indicated amounts of L-Ag473, proteinase K-treated (PNaseK), heated (100°C) L-Ag473 (5 µg/ml). (B) THP-1 cells (2×10^5^/well) were incubated with L-Ag473 (1.5 µg/ml), Pam_3_SK_4_ (100 ng/ml) (InvitroGen, Cat No. tlr1-pms) or LPS (100 ng/ml) (Sigma, Cat No. L4391) alone (−PMB) or pretreated with PMB (+PMB) for 18 hours. For antibody blocking experiments, THP-1 cells were preincubated with the indicated antibody for 30 min before stimulation. IL-8 was undetectable in the untreated cells (−). (C) PBMCs (1×10^6^/well) were incubated with the indicated reagent. TNF-α in the 5-h and 24-hours culture supernatants were determined by ELISA. The result shown is the reading values with the untreated cells as the blank. (D) MoDCs were untreated as control, or incubated with L-Ag473 or NL-Ag473 mutant (100 ng/ml) for 16 hours, and then stained with PE-anti-CD86, FITC-anti-CD80, and PE-anti-CD83 mAbs. The MFI is shown on each picture. The data are representative of three independent experiments.

### Assay for Antigen Presentation

Antigen presentation by matured BMDCs was determined by using an OVA-specific T cell proliferation assay, as described previously [28]. Briefly, BMDCs (2.5×10^4^ cells/well) were incubated with or without L-Ag473 or NL-Ag473 (100 ng/ml) and OVA peptide (2 µg/ml) in 96-well plates (Costar Corning) for 3 hours. CD4^+^ OT-II T cells were added to DC cultures at various DC:T cell ratio as indicated, and then cells were harvested after 72 hours. [^3^H]thymidine (1 µCi/well) was added during the last 16 hours of culture, and the [^3^H]thymidine incorporation was measured by scintillation counting.

### T Cell Priming

Recall assay was performed as described previously [27]. C57BL/6 mice were immunized with incomplete Freund’s Adjuvant (IFA, Sigma-Aldrich) only, IFA + NL-Ag473 (10 µg), or IFA + L-Ag473 (10 µg) via footpad injection. Draining lymph node cells were isolated from immunized mice after 10 days and cultured in 96-well plates at 5×10^5^ cells/well with indicated concentration of Ag473 for 3 days. [^3^H]thymidine (1 µCi/well) was added during the last 16 hours of culture, and the incorporation was measured by scintillation counting. To determine Th differentiation, supernatants were collected from lymph node cell cultures for recall assay as described above. Then, the production of IFN-γ was measured by ELISA kit (eBioscience).

### Cytokine and Chemokine Production

DCs (1×10^6^/ml) were treated with L-Ag473 or NL-Ag473 for 6 hours and 10 µg/ml Brefeldin A (Biolegend) was included in last 4 hours for intracellular cytokine staining. Cells were then blocked with 2.4G2 mAb, stained with anti-CD11c Ab, fixed with 4% paraformaldehyde, permeabilized with Perm buffer (BD pharmingen), and then stained with mAbs to TNF-α, IL-6, and IL-12 p40 (Biolegend), and analyzed by flow cytometry, as previously described [29]. For ELISA, supernatants were collected from 1×10^6^ DCs or macrophages/ml treated with recombinant Ag473 proteins for 24 hours (6 hours for TNF-α and the production of cytokine and chemokine was measured using ELISA kit (eBioscience and R&D system).

### Inactivation of LPS and Ag473

To inhibit LPS activity, the sample was treated with polymyxin B (PMB, Sigma-Aldrich) at 37°C for 30 min. To inactivate Ag473, the protein was treated with proteinase K (2 mg/ml, Promega) at 37°C for 60 min or boiled at 100°C for 60 min.

### Stimulation of Human Monocyte-derived Dendritic Cells (MoDC), Peripheral Blood Cells (PBMC) and THP-1 Cells

Human MoDCs was untreated or treated with L-Ag473 (0–100 ng/ml), NL-Ag473 (100 ng/ml), or LPS (100 ng/ml). The maturation was determined by flow cytometry and TNF-α production was detected by ELISA as described previously [26]. THP-1 cells (5×10^5^ cells/well) in RPMI 1640 medium supplemented with 10% FCS (RPMI/FCS) were stimulated with recombinant Ag473 proteins (0–5 µg/ml), Pam_3_SK_4_ (100 ng/ml), or LPS (100 ng/ml) for 18 hours. For the antibody blocking experiment, THP-1 cells were pretreated with 10 µg/ml of anti-human CD282 TLR2 (BioLegend, Cat No. 309709) or anti-human TLR4 (BD Pharmingen™, Cat No. 551964) (10 mg/ml) for 30 min and then stimulated with LPS or PMB-treated L-Ag473 or Pam_3_SK_4_ (InvivoGen) for 18 hours. Supernatants were collected and the presence of IL-8 was detected using Human IL-8 ELISA Set (BD OptEIA™, Cat# 555244).

## Results

### Assessment of the Purity of the Recombinant Ag473 Proteins

The purities of L-Ag473 and NL-Ag473 were assessed by SDS-PAGE and Western blotting. Silver staining of the gels showed that the NL-Ag473 was purified to apparently homogenous and L-Ag473 proteins contain multi-isoforms (Fig. 1). The slower mobility of NL-Ag473 implicating that L-Ag473 should be correctly processed and the doublet seen in L-Ag473 may represent the apolipoprotein and the mature lipoprotein. Western blot analysis, with mAb 4-7-3 as the primary antibody, indicated that these two proteins essentially contained the same antigenicity.

### The Lipidated Ag473 Possesses Intrinsic Immunogenicity

The effect of lipidation on the immunogenicity of Ag473 protein was further evaluated *in* *vivo*. Balb/c mice were immunized with L-Ag473 (group I), NL-Ag473 (group II), L-Ag473/FA (group III), or NL-Ag473/FA (group IV). The presence of Ag473 specific antibodies was obviously in antisera collected from groups I, III, and IV because strong immunoreactive bands were seen in the corresponding immunoblots, but only a barely detectable band was observed in the blot probed with antiserum from group II mice (data not shown). The specific antibody titers in the pooled sera were further evaluated by ELISA. In these analyses, the anti-L-Ag473 titers were found to be comparable between groups I and III which were ∼2X of the group IV and ∼100X of the group II (Fig. 2A). Collectively, these data indicate that the protein moiety of Ag473 antigen alone is poorly immunogenic, while lipidation on the N-terminus renders this antigen an inherent immunogenicity.

Next we determined the anti-Ag473 IgG subclass distribution by ELISA (Fig. 2B). Substantial levels of the IgG2a and IgG2b in addition to the IgG1 were detected in I, III, and IV groups of mice using 1000-fold diluted sera indicating that both Th1 and Th2 responses were induced. In addition, we found that the Ag473-specific IgG subtype in the 100-fold diluted sera of NL-Ag473-immunized mice (group II) was exclusively IgG1, an indicative isotype of Th2 response. These data suggest that the lipid moiety of Ag473 not only possesses an immunopotentiating activity but also has the ability to modulate the type of immune response.

### L-Ag473 Induces Mouse BMDC and Macrophages to Produce Cytokine and Chemokines

DCs residing in most tissues in an immature state are an early encounter linking innate and adaptive immunity. Activated DCs secrete cytokines and interact with lymphocytes to initiate and shape the adaptive immune response. To investigate the molecular mechanism underlined the self-adjuvanting activity of L-Ag473, we examined the capacity of Ag473 proteins to induce the expression of TNF-α, an important autocrine stimulator of dendritic cells. While no effect was observed for NL-Ag473 with concentration up to 100 ng/ml, L-Ag473 induced the expression of TNF-α (Fig. 3A) in a dose-dependent manner (Fig. 3B) indicating the importance of the lipid moiety and also provided an indirectly evidence that the observed effect of the L-Ag473 was not caused by contaminated LPS. L-Ag473 also promoted the TNF-α production by macrophages (Fig. 3C), suggesting that L-Ag473 is a common stimulator for APCs.

LPS are heat stable and PMB can fully neutralize *E. coli* LPS [30]. To further demonstrate that the biological activity observed in L-Ag473 is not caused by LPS, we compared the effect of PMB, heat, and proteinase K treatments on the ability of L-Ag473 and LPS to induce TNF-α expression in BMDCs (Fig. 4). While the pretreatment with PMB abolished the LPS activity, L-Ag473 activity was only slightly reduced. In addition, we found that proteinase K digestion and heat treatment abolished the induction of TNF-α expression by L-Ag473 but did not affect the LPS activity. These results strongly indicate that the activation of BMDC is an intrinsic property of L-Ag473 and this activity is conformational dependent.

Next, we measured the levels of proinflammatory cytokines and chemokines in the culture supernatants by ELISA. As shown in Fig. 5, BMDCs produced TNF-α, IL-6, IL-12 p40, MCP-1 (CCL2), MIP-1 (CCL4), and RANTES (CCL5) after L-Ag473 treatment in a dose-dependent manner and no effect was seen for NL-Ag473 (data not shown). Collectively, these data demonstrate that L-Ag473 can potentially activate DCs and enhance innate immune response.

### L-Ag473 Promotes BMDC Maturation

To determine whether L-Ag473 treated DCs are matured into effector DCs, we examined the expression of surface molecules on BMDCs after treating with L-Ag473 for 16 hours (Fig. 6). The levels of class II MHC molecule as well as the costimulatory molecules CD40, CD80, and CD86 on the L-Ag473 treated-DCs were greater than that of cells incubated with NL-Ag473 and the medium alone (Fig. 6A). In addition, like the LPS-treated DCs, the uptake of dextran in L-Ag473-treated DCs was significantly decreased compared with the untreated or NL-Ag473-treated DCs (Fig. 6B). Furthermore, the L-Ag473-treated DCs efficiently presented OVA peptides and activated OT-II T cells (Fig. 6C). Together, these data demonstrated that L-Ag473 has an ability to induce BMDC maturation.

### Ag473 Induces Specific T Cell Activation in vivo

The primary function of mature DCs is to induce T cell activation and proliferation. To determine the functional consequences of L-Ag473 induced DC maturation, we performed recall assay to evaluate the effect of L-Ag473 on T cell priming *in vivo*. C57BL/6 mice were immunized with incomplete Freund’s adjuvant (IFA), L-Ag473 or NL-Ag473 via footpad injection. Draining lymph node cells were isolated after 10 days and cultured in the presence of L-Ag473 for 3 days. Consistently, cells isolated from L-Ag473-immunized mice showed significant proliferation compared to cells from control or NL-Ag473-immunized mice (Fig. 7A). These data reveal that L-Ag473-treated BMDCs induce Ag-specific T cell priming and proliferation *in vivo*. In addition to proliferation, significant higher levels of IFN-γ were detected in the culture supernatants of L-Ag473-primed compared to control (IFA alone) or NL-Ag473-primed lymph node cells (Fig. 7B). Together with the detection of IL-12 in L-Ag473 treated BMDC (Fig. 5), we conclude that L-Ag473 has an ability to induce polarization of T cells to the Th1 phenotype.

### TLR4 is Involved in L-Ag473 Induction of Mouse BMDC Maturation

It is well accepted that bacterial lipoproteins are recognized by TLR2 cooperated with TLR1 or TLR2/6 dependent mainly on the N-terminal lipidated structures. This conclusion is drawn based mainly on the studies with synthetic lipopeptides [31]. However, it is still possible that different TLR profiles were required for the recognition between different lipoproteins. Thus, we examined the stimulation of L-Ag473 on DCs derived from TLR2^−/−^ mice first, with LPS and Pam_2_CSK_4_ as the controls. Unexpectedly, while no response was obtained with Pam_2_CSK_4_ treatment, TNF-α production and the expression of CD86 and CD40 were only slightly reduced in TLR2-deficient DCs stimulated by L-Ag473, which was similar to LPS treatment (Fig. 8A, left panel). Consistently, responses of MyD88^−/−^ DCs to L-Ag473 and LPS stimulation were similar (Fig. 8B). These results imply the involvement of TLR4 in L-Ag473 recognition because of its partial dependency of MyD88. Thus, we examined the TLR4 mutant DCs in response to L-Ag473 stimulation. We found that TNF-α production and DC maturation were diminished more dramatically than that in TLR2^−/−^ cells (Fig. 8A, right panel). The results thus suggest that TLR4 is involved in L-Ag473 induction of mouse BMDC activation.

### Effects of Ag473 on Human Cells

To further validate the above observation and as an initial attempt to test if L-Ag473 has any immunomodulating activity in human, we used the same batch of L-Ag473 to treat human THP-1 cells. The production of IL-8 induced by L-Ag473 was dose-dependent (Fig. 9A) and this activity was dramatically reduced when L-Ag473 was treated with proteinase K or 100°C for 1 hour and in the presence of anti-TLR2 (Fig. 9B). These results indicate that L-Ag473 is able to activate THP-1 cells in a TLR2-dependent manner and further confirmed that the immunomodulating activities observed in the purified L-Ag473 is not due to the LPS contamination. The effects of L-Ag473 on human PBMC and MoDC were then examined. Significantly higher levels of TNF-α were detected in the supernatants from the L-Ag473 treated PBMC (Fig. 9C) and MoDC (data not shown). Furthermore, the levels of CD80, CD83, and CD86 were higher in L-Ag473 treated MoDC than in the NL-treated and untreated MoDC (Fig. 9D). Collectively, the results demonstrated that L-Ag473 is a strong immunostimulator for human APCs.

## Discussion

The availability of genome sequence has resulted in a fruitful success in subunit vaccine development in terms of target identification. However, the immunogenicity of the antigen alone may not be sufficient to confer effective protection. Currently the only adjuvant approved for human use in the United States and most countries is aluminum salt which is comparatively weak and only work with certain diseases. This study demonstrated that the *E. coli* produced meningococcal Ag473 lipoprotein is able to induce an antibody response comparable with the presence of external adjuvants (Fig. 2), indicating that Ag473 lipoprotein possesses self-adjuvanting activity.

Like most characterized lipoproteins, the lipid moiety of the Ag473 protein is required for the self-adjuvanting activity and this property was attributed to its ability to activate and promote DC maturation (Fig. 6). The detections of IL-12 in L-Ag473 treated DCs (Fig. 5) and IFN-γ in the recall assays (Fig. 7B) indicating that L-Ag473 has an ability to skew the immune response toward Th1 response. This is in consistence with the *in vivo* antibody response which showed that substantial levels of IgG2 were detected in the L-Ag473 immune serum in addition to the IgG1 (Fig. 2). The ratios of IgG1/IgG2 in the L-Ag473 (with or without FA) and NL-Ag473 immune sera were approximately 0.75 and 2, respectively. Taken into consideration that Balb/c mice are prone to produce Th2 response and L-Ag473 immunized Balb/c mice could be protected from a lethal meningococcal infection in the absence of complement –mediated bactericidal activity [21], our data indicate that L-Ag473 has an intrinsic immunogenicity with capability to induce both humoral and cell-mediated immune responses (Fig. 3 and 5). The observation that L-Ag473 stimulates human blood monocytes to produce TNF-αand promotes MoDC maturation (Fig. 9) suggests a great chance that L-Ag473 is immunogenic in human.

It is well known that the activity of *E. coli* LPS can be neutralized by PMB and resistant to heat and proteinase K treatments. Our results show that L-Ag473 activity was not affected by PMB but reduced after heating and proteinase K digestion. In addition, as NL-Ag473 failed to induce TNF-α production (Fig. 3A), we compared the effect of NL-Ag473 spiked with LPS in the presence or absence of PMB on induction of TNF-α production. TNF-α production was induced in NL-Ag473/LPS treated cells, but such activity was completely blocked in the presence of PMB (Fig. 9C). Collectively, we believe that the observed immunomodulatory activity is an inherent property of L-Ag473 but not due to LPS containment in the preparations.

Although L-Ag473 has the capability to activate and promote mouse and human dendritic cell maturation, the outcome of immune responses may be different as different TLR signaling pathways were found to be involved in these two species. TLR2 has been recognized as the primary receptor for bacterial lipoproteins/lipopeptides, synthetic peptides and bacterial lipoproteins [18], [32]. As seen in most bacterial lipoproteins, TLR2 is involved in the L-Ag473 activation of human monocytic cells (Fig. 9B). However, we found that the responses of TLR2^−/−^ and wild type BMDC to L-Ag473 stimulation were comparable but dramatically reduced in TLR4-defective DCs (Fig. 8A) suggesting that TLR4 is the primary receptor for L-Ag473. This is not unprecedented since lipoproteins BmpA of *Borrelia burgdorferi* and Omp16 of *Brucella abortus* were reported to signal via TLR4 and such property is attributed to the protein portion [19], [20]. Noteworthy, the protein portions of the above two lipoproteins are recognized by TLR4. The observation that NL-Ag473 (Fig. 3A), heat-treated, or proteinase K-treated L-Ag473, failed to induce the production of TNF-α by BMDCs (Fig. 4) suggesting that L-Ag473 signaling via TLR is dependent on certain conformation formed by both the lipid moiety and the protein moiety. Recently, a triacylated hybrid lipoprotein (rlipo-D1E3) with N-terminal 23 amino acid residues identical to the L-Ag473 has been shown to activate BMDCs via TLR2 signaling using either TLR1 or TLR6 as a co-receptor [23], [33]. This co-receptor usage may be attributed to its lipid moiety which was deciphered to contain a C16:1 in addition to two C16:0 fatty acid molecules. Accordingly, the difference in the TLR usage between the L-Ag473 and r-Lipo-D1E3 implicates that the lipidated N-terminal 23-amino acid region is not sufficient to form the TLR4 binding domain for triggering a TLR4-mediated immunological pathway in mouse. Similar to LPS molecules, bacterial lipopropteins may assume various supramolecular structures which may affect the TLR dependence [34]. Therefore, we cannot rule out the possibility that L-Ag473 may also signal via other molecular mechanisms.

Differential TLR recognition of leptospiral lipid A and LPS in murine and human cells has been proposed to represent an escape mechanism from human cell innate immune recognition [35]. As human nasopharynx is the sole natural habitat for *N. meningitidis,* it is expected that the innate immune responses against this bacterium in human and mouse may be different. The current results suggest that Ag473 may be one of the molecules that participating in shaping this species-differential response; however, this assumption await to be tested. Nevertheless, the induction of IL-8 production in THP-1 cells by L-Ag473 was also conformational dependent because the induction of IL-8 production was diminished in the heated-treated L-Ag473 and abolished in proteinase K treated L-Ag473 (Fig. 9). Like in BMDC, the effect of proteinase K treatment was more profound than by heating. Furthermore, the observed stimulatory activities of heated L-Ag473 in human THP-1 cells (33% of the L-Ag473) (Fig. 9) were higher than in mouse DCs (15% of the L-Ag473) (Fig. 4). Previous biochemical/biophysical characterization indicated that L-Ag473 can regain its immunological properties after heating, lyophilized or frozen [22]. As the cytokine productions by THP-1 cells and DC cells were measured after 18 hours and 6 hours post-treatment, respectively, variation in the amounts of refolded L-Ag473 may be one reasonable explanation.

In conclusion, this study describes, for the first time, the immunomodulatory activity of a meningococcal lipoprotein in mouse and human cells. The data indicate that the overall conformation of L-Ag473 is required for its immunomodulatory activity and this protein may play a role in shaping of differential response against meningococcal infection in human and mouse. Whether this is unique to L-Ag473 or common to other lipoproteins with unusual primary structures (e.g. no aromatic residue, alanine rich, and contain tandem repeats) remained to be determined. The results presented here also suggest a new paradigm for the ligand-TLR interaction in addition to demonstrating the self-adjuvanting activity of the vaccine candidate L-Ag473. Together with the findings that ag473 gene (NMB1468) was upregulated during infection [36], [37], the properties of self-adjuvanting and activating human immune cells enhance the feasibility and effectiveness of using L-Ag473 as a vaccine antigen in human.
